# Psychological Readiness for Surgery Through Prehabilitation and Treatment: A Longitudinal Qualitative Study

**DOI:** 10.1002/pon.70594

**Published:** 2026-08-28

**Authors:** Aniqa Hussain, Thomas Schroder, Mike Rennoldson, Michael Baliousis

**Affiliations:** ^1^ University of Lincoln Lincoln UK; ^2^ Lincolnshire Partnership Foundation NHS Trust Lincoln UK; ^3^ University of Nottingham Nottingham UK; ^4^ Nottingham University Hospitals NHS Trust Nottingham UK

**Keywords:** adult, anxiety, cancer, clinical psychology, motivation, neoplasms, oncology, prehabilitation, preoperative exercise, surgery

## Abstract

**Background:**

Psychological prehabilitation aims to prepare patients for cancer treatment, yet psychological readiness is under‐specified and distress during and after treatment is common. A patient‐informed account of readiness is needed to guide readiness‐focused, proactive psychological support in cancer care.

**Aims:**

To examine patients' experiences of psychological readiness for cancer surgery across the prehabilitation–treatment pathway.

**Methods:**

We used a longitudinal qualitative design. Ten adults enroled in a tertiary cancer prehabilitation service for major elective surgery completed semi‐structured interviews before surgery (T1) and after surgery (T2). We analysed data using Reflexive Thematic Analysis with an inductive, semantic approach, and compared change and stability across time points using a longitudinal analytic matrix.

**Results:**

Participants described psychological readiness as developing a future‐focused “mindset to have surgery”, comprising three interrelated elements that were stable from T1 to T2: confidence, motivation, and contained anxiety, that is, anxiety that remained expected and manageable rather than absent or overwhelming. Three processes shaped this mindset. **Reliable and complete information** from professionals and credible peers supported clearer and more concrete expectations, increased confidence, and helped keep anxiety manageable. Participants preferred candid information about plausible difficulties over reassurance that downplayed them. **Reciprocal** **support** from family, peers, and professionals strengthened motivation and reduced loneliness. Readiness could be held back when participants found it difficult to tell loved ones about their diagnosis. **Agency** developed through drawing on personal resources and taking controllable steps, including making progress visible through prehabilitation activity and staff encouragement.

**Conclusions:**

These findings identify provisional, patient‐informed targets for psychological preparation within multimodal prehabilitation. They provide a foundation to operationalise and measure readiness and, following work on transferability, co‐production and feasibility, to test whether readiness‐focused support in prehabilitation is associated with post‐operative adjustment.

## 1 Background

Up to half of cancer patients experience emotional distress (e.g., anxiety, depression, fear of progression, general distress) during treatment [1, 2, 3], which increases suicide risk and undermines adherence, recovery, survival, and quality of life, adding, in US estimates, over $17,000 per patient annually to healthcare costs, and disproportionately affecting socially marginalised groups [1, 4, 5, 6]. Psychological interventions are less readily accepted once distress has manifested or treatment has started [7], as treatment side‐effects (e.g., fatigue, nausea, immunosuppression) and logistical demands limit capacity to engage [8, 9]. As a consequence, prevention has become a clinical and policy priority nationally and internationally [10, 11, 12, 13].

One promising approach is prehabilitation. Recent clinical and implementation guidelines define prehabilitation as “a needs‐based multimodal intervention, before and during cancer treatment”, which aims to “optimise physical, nutritional and psychological status, enhance readiness for and tolerance of treatments, and improve recovery and/or quality of life” [14]. Preparation is therefore best begun soon after diagnosis, but is not confined to the interval before surgery: where the pathway from diagnosis to first treatment is short, or surgery follows neoadjuvant treatment, readiness may need to be revisited at several points. Prehabilitation is increasingly seen as best practice and has been effective in improving treatment outcomes and reducing complications [14, 15, 16, 17].

Yet prehabilitation programmes remain weighted towards physical optimisation. Exercise and nutritional components have clear proximal targets—such as fitness, strength, nutritional markers—which translate into better post‐operative functioning and recovery [18]. By contrast, psychological preparation is less clearly specified: patients may receive broad self‐help materials when overwhelmed, or targeted input once distress is evident, rather than readiness‐focused support designed to reduce the risk and impact of treatment‐related distress and to support adjustment [19, 20, 21]. This reflects a more fundamental problem: we do not yet have a clear, patient‐based understanding of what it means to be psychologically prepared for cancer treatment. Without this, preventative psychological care lacks a defined target.

There are clues about what psychological readiness might involve. In sports psychology, readiness to return to sport after injury comprises appraisals (e.g., confidence), feelings (e.g., anxiety), and behaviours (e.g., approach/avoidance) [22]. Similarly, resilience theory and prospective longitudinal studies of psychological predictors of distress in cancer suggest that both personal and relational mechanisms shape how people respond to challenges. At a personal level, these include how patients interpret illness, what they expect, how they cope, and how they regulate thoughts and emotions [23, 24]. At a relational level, the availability and quality of social support, loneliness, and coping within close relationships seem important [1, 23, 24]. These findings point to plausible psychological targets.

But these findings do not define what psychological readiness means to cancer patients. Without a patient‐informed account of readiness, we cannot specify what psychological prehabilitation should change in patients' experience, design appropriate programmes, or evaluate whether they prepare cancer patients for treatment effectively.

To address this gap, we examined patients' experiences of psychological readiness for cancer treatment across the prehabilitation‐treatment pathway. Consistent with the remit of the study service, we focussed on patients preparing for cancer surgery, while recognising that psychological readiness is likely to be relevant across the treatment pathway, and explored.

- What meanings, emotions, and expectations constitute psychological readiness for cancer surgery;
- How psychological readiness changed or remained stable from prehabilitation to early post‐operative recovery;
- What contexts, resources, and processes patients perceived as shaping readiness, including perceived supports within current prehabilitation.

## 2 Method

### 2.1 Design and Materials

We adopted a critical realist epistemology and used a longitudinal qualitative design [25, 26, 27]. We conducted semi‐structured interviews at two time points: before surgery (T1) and after surgery (T2). At T1, we explored participants' experiences of psychological readiness during prehabilitation prior to surgery. At T2, we examined how their perspectives on readiness had changed following surgery. This design allowed us to examine how participants understood psychological readiness and how these understandings evolved across the prehabilitation–treatment pathway.

We developed two semi‐structured interview guides (pre‐ and post‐surgery; Supporting Information S1) iteratively with input from the study's patient advisor. We grounded the guides in the study aims and in literature on personal and relational mechanisms relevant to psychological adjustment in cancer [1, 23, 24]. We also discussed the topic areas with prehabilitation professionals identified through clinical networks. We finalised both guides before recruitment opened; neither was redesigned in response to emerging themes. We used them flexibly rather than as fixed schedules. After the first interview, AH changed how she introduced the format and prompted, but not the topic areas.

At T1, we completed a proforma to capture personal and clinical characteristics for descriptive purposes. We did not administer quantitative measures of psychological wellbeing because the study aimed to characterise experiences of readiness, not to predict or categorise post‐operative adjustment.

The NHS Nottingham 1 Research Ethics Committee reviewed the study, and the Health Research Authority approved it (Ref: 24/EM/0129).

### 2.2 Participants

Participants were eligible if they were: (a) 18 years or older; (b) enroled in cancer prehabilitation; (c) scheduled to undergo major elective cancer surgery requiring inpatient admission and, in some cases, post‐operative high‐dependency or intensive care within 6 months; (d) able to participate in an interview in English; and (e) able to provide informed consent. We aimed to interview approximately 10 participants at both time points, seeking data sufficiency [28, 29].

We used purposive sampling [30] to recruit participants from a tertiary prehabilitation service in England serving a regional population of approximately 5.4 million people. The service provides prehabilitation for patients undergoing major cancer surgery and does not support non‐surgical treatment pathways. It supports communities characterised by high ethnic diversity and significant socioeconomic deprivation. The programme adheres to the national prehabilitation framework [14] and focuses on structured exercise, healthy lifestyle advice and psychological wellbeing support. Physiotherapists and exercise professionals deliver the programme and receive training and supervision to provide first‐line psychological support. The service also provides onward referral to psychological professionals for patients with more complex needs and is supported by consultant anaesthetists and paid prehabilitation link workers, employed by a partner voluntary‐sector organisation, who offer additional emotional, practical, financial, spiritual and social support and who have completed training in first‐line psychological support.

### 2.3 Procedure

We recruited participants between December 2024 and February 2025 using two approaches. First, members of the prehabilitation team introduced the study to eligible patients during their initial prehabilitation assessment. Patients who expressed interest provided permission for the research team to contact them. Second, members of the research and prehabilitation teams shared study invitations during prehabilitation exercise sessions, and interested patients contacted the research team directly. AH followed up and completed informed consent procedures with those who agreed to participate.

AH conducted all interviews with participants individually by telephone or video call, according to participants' preference. AH audio‐recorded each interview and made field notes during interviews to capture emerging topics for follow‐up prompting. Because the subject matter could be distressing, AH reminded participants that they could decline any question, take a break, or stop the interview at any point. She monitored for signs of distress, could signpost participants to support, and provided a debrief with written support information after each interview.

We conducted pre‐surgery interviews between January and February 2025, as soon as practicable following enrolment. We conducted post‐surgery interviews between February and April 2025. We scheduled these around participants' recovery and readiness to take part. We did not record dates of surgery for research purposes, so we cannot report the interval between surgery and interview; we note this as a limitation.

### 2.4 Data Analysis and Reflexivity

AH transcribed all interviews using the University of Nottingham's automated transcription software and checked each transcript for accuracy. AH analysed the data using Reflexive Thematic Analysis (RTA) [31], adopting an inductive and semantic approach consistent with a critical realist epistemology. For the RTA, AH familiarised herself with the data, generated initial codes, developed candidate themes, reviewed and refined themes, and defined the final thematic structure. AH used visual mapping and written analytic notes to support theme development.

In line with RTA principles, we conceptualised themes as interpretative constructions rather than discoverable entities and therefore did not seek inter‐rater reliability. Instead, we emphasised reflexivity, transparency and iterative coding and team discussion to support analytic rigour [31].

To examine longitudinal change, AH compared T1 and T2 data both within and between participants [32]. She organised candidate themes into a comparative matrix to explore continuity and change across time points. Throughout analysis, AH returned to the original transcripts to ensure that themes remained grounded in participants' accounts.

The research team met regularly to review codes and candidate themes. These discussions supported reflexive interpretation and theme refinement. AH maintained a reflexive record throughout the process [31].

### 2.5 Research Team

AH (female) conducted the study as part of a doctoral programme in Clinical Psychology. She had prior training in qualitative research methods and no personal experience of cancer or prehabilitation. She had no prior relationship with participants, and contact was limited to recruitment and interviews. Participants were informed of AH's role as a doctoral researcher, and no therapeutic role was adopted.

AH conducted the study under supervision from MB, TS and MR (males), senior clinical academics trained to doctoral level as psychological practitioners and working within NHS services. MB and MR work in cancer psychology; TS works in psychotherapy with expertise in adjustment to adversity.

We considered potential influences on interpretation, including AH's indirect family experience of cancer and the team's prior engagement with prehabilitation literature, and addressed these through reflexive discussion during analysis.

## 3 Results

### 3.1 Participant Characteristics

Of the 11 patients who expressed interest in the study, 10 participated; one withdrew before the first interview due to time constraints. Of the 10 final participants (five female), all completed interviews at both T1 and T2. T1 interviews ranged from 13 to 40 minutes, and T2 from 11 to 31 minutes. Some interviews were brief. We judged the dataset adequate against the focussed study aim and the paired longitudinal design, while recognising that brevity may have constrained the depth of some accounts. Participants had a mean age of 64.3 years (range 35–84). The sample was predominantly White British (90%), with one British Pakistani participant. Most participants (80%) were retired; one was a full‐time carer and one worked in healthcare administration. Seventy percent had completed school‐level education and 30% held a degree or higher qualification. Index of Multiple Deprivation data were available for nine participants: 30% lived in the most deprived deciles [1, 2, 3], 30% in the least deprived [8, 9, 10].

Table 1 summarises cancer diagnoses and engagement with prehabilitation. Colorectal cancer was the most common diagnosis (80%). Most participants (90%) received a targeted prehabilitation programme. None were referred for specialist psychological support (by psychological professionals) during prehabilitation. All participants received routine psychological advice embedded within the programme.

**TABLE 1:** Participant cancer site, prehabilitation programme and engagement.

Participant (pseudonym)	Cancer site	Exercise sessions attended	Pre‐surgery time in prehabilitation (days)
Jane	Colorectal	0^a^	9
Jack	Upper gastrointestinal	21	82
Sophie	Colorectal	5	40
Selina	Colorectal	11	49
Moin	Colorectal	1 (+ 3 support calls)	43
Ben	Colorectal	3	18
Anna	Colorectal	19	174
Adam	Colorectal	6	17
Liam	Thoracic	0^b^	35
Hannah	Colorectal	9	22

^a^ Unable to attend exercise sessions due to time constraints (surgery < 2 weeks from diagnosis) but received twice‐weekly practical and emotional support via telephone calls.

^b^ Did not attend exercise sessions due to feeling unwell.

### 3.2 Themes

**FIGURE 1:**
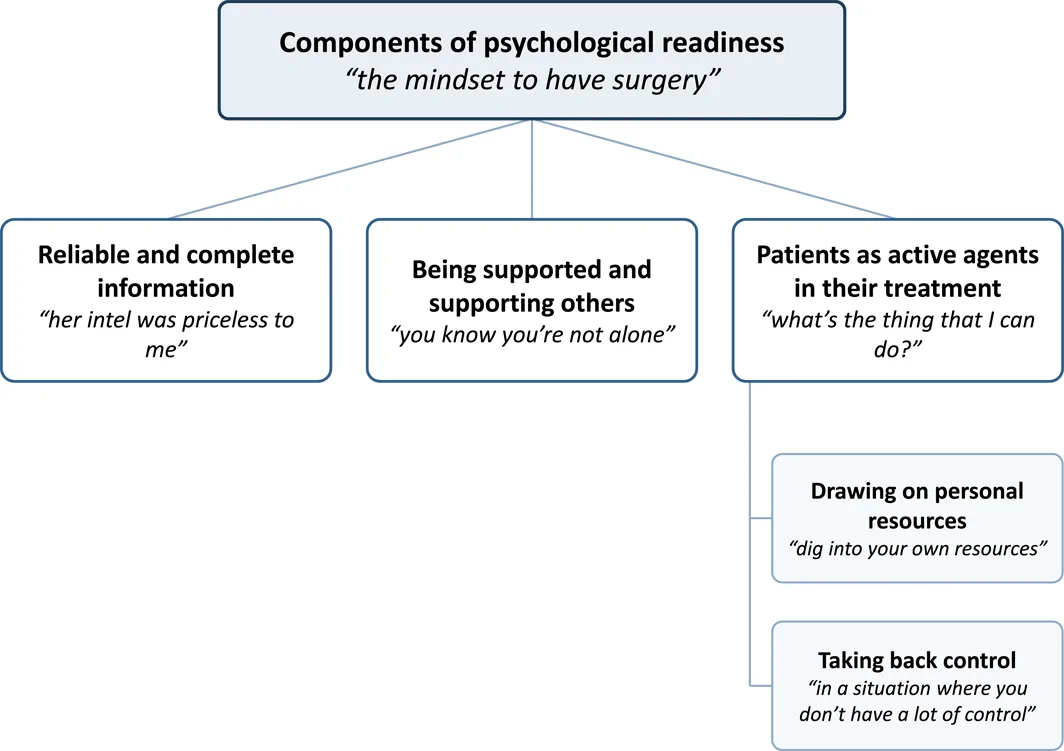
Psychological readiness, the “mindset to have surgery”, was shaped by three processes: reliable and complete information; being supported and supporting others; and patients as active agents in their treatment. The third comprised two subthemes: drawing on personal resources and taking back control.

We generated one overarching theme, three main themes, and two subthemes (Figure 1). The overarching theme, *“the mindset to have surgery”*, captured participants' descriptions of psychological readiness as comprising three interrelated elements: confidence, motivation, and contained anxiety—that is, anxiety that remained expected and manageable rather than absent or overwhelming, and that did not prevent participants from proceeding with surgery. The three main themes describe how this mindset developed. First, access to complete and reliable information supported confidence and helped keep anxiety manageable. Second, support from family, peers, and professionals strengthened both confidence and motivation. Third, participants described the role of becoming active agents in their readiness, drawing on personal resources and taking back control. Prehabilitation was interpreted as a key context within which these processes unfolded.

### 3.3 Thematic Map

#### 3.3.1 Components of Psychological Readiness: “The Mindset to Have Surgery”

Participants described psychological readiness as developing the “mindset to have the surgery” (Anna, T1). This mindset comprised three interrelated elements: confidence, motivation, and contained anxiety. Participants' accounts of these components were stable from T1 to T2.

Confidence referred both to understanding what to expect from treatment and to believing in one's ability to cope with physical changes following surgery. Receiving reliable information enhanced understanding, while engaging in prehabilitation and feeling supported by professionals strengthened confidence in coping.

I'm getting more confident in myself and when the time comes, I’ll be [mentally] ready.

(Jack, T1)

Motivation reflected participants' drive to engage with treatment in order to recover more quickly and return to “normal life” activities (Sophie, T2). Family support was a key source of motivation. The possibility of improved recovery through prehabilitation encouraged participants to attend sessions and position themselves as active agents in their treatment.

[Psychological readiness is] motivation.

(Hannah, T2)

I’ve gotta get to a point where I can manage to look after them [grandkids]. I’m well enough.

(Hannah, T2)

*“Contained anxiety”* (Selina, T1) captured the idea that readiness did not require the absence of anxiety. Participants described anxiety as inevitable but manageable. Preparation involved normalising and reducing anxiety, and keeping it manageable enough to proceed with surgery. Keeping anxiety manageable was supported by drawing on personal resources, engaging with support, and accessing reliable information.

Nervousness is part of the readiness.

(Sophie, T1)

…being mentally prepared, as you can be, and reducing your anxiety…I am bound to have some anxiety levels though.

(Jane, T1)

The following themes further unpack how these components developed and were shaped across the prehabilitation–treatment pathway.

#### 3.3.2 Reliable and Complete Information: “Her Intel Was Priceless to Me”

Participants described reliable and complete information as central to developing psychological readiness. Information from trusted professionals and peers helped them understand what to expect, strengthening confidence and supporting “contained anxiety”. In contrast, participants viewed “Googling” as unreliable and potentially anxiety‐provoking.

For most, cancer diagnosis and treatment were novel experiences that came with uncertainty about what to expect. Two participants (Sophie and Jack) had previously received cancer treatment and felt they “roughly knew what to expect” (Sophie, T2), which supported their readiness this time. More commonly, participants reduced uncertainty by seeking information from trusted sources, including clinical teams and people who had undergone similar treatment.

The phone calls I've had and the appointments that have come…and the information I've been given is very, very good… it prepares you.

(Jane, T1)

Participants turned to peers with lived experience to understand “what to expect” and to feel “mentally ready” (Sophie, T1). Ben described a friend who had undergone the same surgery walking him through it “step by step”, while Moin sought peer support through a Maggie's Café—a drop‐in space in a Maggie's centre, a charity providing free practical and emotional support to people affected by cancer—and valued the accuracy of what he was told in hindsight. These accounts came from participants' own networks and from people they met in cancer support settings, not from peer support arranged by the clinical team. Participants judged them credible when they were specific and closely matched their own treatment; at T2, some also judged credibility by how well an account matched what they had since experienced.

A friend of mine who actually had the same surgery as me… she walked me through step by step…her intel was priceless to me.

(Ben, T2)

She was clear about everything…exactly what she said is what I experienced.

(Moin, T2)

Participants were cautious about “Googling”, describing it as uncontained and difficult to regulate. They worried it could escalate anxiety and expose them to information of uncertain quality. Some participants felt able to search safely—for example, where they or their family members had a healthcare or academic background—and described being “confident about looking things up” (Hannah, T1) without exacerbating anxiety. One participant reflected on the “unhelpfulness of people without qualifications” (Liam, T1) providing them with advice and suggested that this hindered their psychological readiness.

There’s Google, I can look at it but there’s so many things you can find, it’s better when you get information from the person, someone who has gone through it or treats people.

(Moin, T1)

For some participants, judgements about whether they had been psychologically ready shifted from T1 to T2 when surgical outcomes deviated from what they had been told. Moin recalled asking whether a stoma was possible and being repeatedly told “no”, but waking with a stoma, which affected his trust and left him feeling it would be “hard to trust next time” (Moin, T2). In contrast, Hannah valued being warned that things might not go as expected; this candid framing strengthened her confidence in the team's plan.

They also warned me that things might not be as I expected… they had prepared me for that…I felt confident in what they’re saying they’re going to do because of the way they presented it to me.

(Hannah, T2)

Participants' accounts suggested that complete and candid information was valued over reassurance that sought to minimise distress. Such transparency helped keep anxiety manageable. Jane (T2) described a difficult pre‐operative appointment in which the consultant explained “everything that could go wrong”. She reported a brief “wobble… it was over within minutes”, recognised this anxiety as “to be expected”, and emphasised that “they have to tell you to prepare you”. These accounts suggest that preparedness depended less on being assured of a good outcome than on expectations that allowed for plausible deviations. Unexpected outcomes were most disruptive when participants had been told they would not occur.

#### 3.3.3 Being Supported and Supporting Others: “You Know you're Not Alone”

Participants described social support as a key contributor to psychological readiness. Support from family and friends provided practical and emotional help and strengthened motivation to engage with treatment. Support from peers and professionals, including within prehabilitation, helped build confidence and reduced isolation.

Family support enhanced readiness by offering reassurance, practical help, and reasons to get through treatment and “just get better” (Sophie, T2). Participants described drawing on wider networks when needed.

I’ve got family, friends and neighbours I can go and talk to.

(Sophie, T1)

At T1, some participants felt their readiness was “on hold” until they had disclosed their diagnosis to loved ones. They wanted to protect others from “distress and burden” (Hannah, T1), but described limited confidence or skills for having these conversations. Readiness increased when participants could speak openly—sometimes lightly—about the diagnosis, which created shared understanding.

I knew that I’d have to tell people, I found that hard…I just don’t think the staff have time for things like that.

(Hannah, T1)

We’ve brought it out into the open, bringing it out into the open has given us all greater understanding.

(Ben, T1)

Participants also described prehabilitation as reducing a “lonely experience” (Jack, T1) by providing contact with both professionals and peers. Sessions were described as enjoyable and offered opportunities to “have a laugh” (Jane, T1) and build rapport.

It was good, you know, we got a bit of a rapport going…I enjoyed it.

(Selina, T1)

Moin, who could not attend exercise classes, had limited access to peer support within prehabilitation and sought support instead through a Maggie's Café.

Finally, participants described how encouragement from prehabilitation staff strengthened confidence, including confidence in physical capability. Perceiving that staff were confident in them appeared to reinforce their own confidence.

Because they [prehab professional] feel confident, I feel confident.

(Hannah, T2)

#### 3.3.4 Patients as Active Agents in Their Treatment: “What's the Thing That I can do?”

Participants described psychological readiness as involving active engagement in their treatment rather than passive waiting. They reflected on what they brought to the diagnosis and surgical pathway, and how drawing on personal resources and taking practical steps to regain control strengthened confidence and helped keep anxiety manageable.

### 3.4 Drawing on Personal Resources: “Dig Into Your Own Resources”

Ben (T2) reflected that prehabilitation encouraged people to “dig into your own resources”. Participants described drawing on resources they had developed throughout life, such as their ability to compartmentalise, practical nature, or faith.

I’m a practical person…I’m capable of compartmentalising so I can cope.

(Jane, T1)

Only listening to the Quran [helped reduce anxiety]. I just put my headphones on, listen to the Quran and walk around the park.

(Moin, T2)

For some, T2 reflections suggested that previous coping skills alone were not always sufficient for surgery and recovery. Engagement with prehabilitation appeared to extend or strengthen these existing resources. In contrast, Liam described not prioritising prehabilitation during a busy period and later questioning that decision.

I’ll be honest, fitness was mentioned but you think you’re alright, 70 years old and can move your legs and hips but…now I think preparation is the most important part.

(Liam, T2)

### 3.5 Taking Back Control: “In a Situation Where You don't Have a Lot of Control”

Participants described surgery as a context of limited control. Readiness involved identifying actions within their control. For Liam, managing anxiety through medication was one such step.

To be mentally ready…I've got medication now to calm down me anxiety.

(Liam, T1)

Prehabilitation provided structured opportunities to act. Participants linked improving physical fitness to greater control over “better outcomes and quicker…recovery” (Hannah, T1), which reinforced confidence in coping.

In a situation where you don't have a lot of control, it [prehabilitation] feels like it gives you some of that control back, well, what's the thing I can do?.

(Hannah, T1)

At T2, participants reflected on this active engagement as part of what helped them approach surgery and interpret recovery. Perceived progress in fitness, alongside learning and practising specific exercises, strengthened their sense of agency and confidence in their body's ability to cope with surgery and recovery.

Prehab definitely helped…I was so weak afterwards and you’ve got no strength which makes you wonder if it did. But I think if I didn’t, I would have felt even worse.

(Anna, T2)

100% without a doubt I think it’s such an important part of going into surgery…I knew my body could cope with what I’ve had.

(Ben, T2)

## 4 Discussion

Prehabilitation aims to “prepare” patients for treatment, but psychological readiness is not clearly defined from the patient perspective [14]. In this longitudinal study, participants described readiness for cancer surgery as a future‐focused “mindset to have surgery” comprising confidence, contained anxiety, and motivation to engage with preparation. Readiness did not mean eliminating anxiety; participants described it as expected but manageable. This matters clinically: psychological preparation need not remove normal anxiety, and the target is anxiety that remains manageable and compatible with approaching treatment, alongside confidence and motivation. This resembles sports psychology accounts of readiness to return to sport after injury, which similarly integrate appraisals, affect, and approach or avoidance behaviour [22], while the clinical context and aims differ. Participants emphasised three routes through which readiness developed—reliable information, reciprocal support, and agency—which help specify practical targets for preventative psychological care within multimodal prehabilitation.

Participants' emphasis on information extends existing work on information needs and uncertainty by distinguishing between reassurance and preparation. Participants valued complete and candid information that helped them anticipate what recovery might involve, even when it temporarily increased anxiety. Peer accounts were particularly influential when they were concrete and credible. These accounts came from participants' own networks and from cancer support settings, not from peer support arranged or moderated by the clinical team; participants judged them credible when they were specific and closely matched their own treatment. Affective forecasting theory suggests that expectations about future emotional experiences can have downstream implications for adjustment, and that such expectations are shaped by the information and narratives people draw on [33, 34]. In our data, the credibility and completeness of information shaped participants' expectations and their sense of preparedness. This raises a testable question: whether expectation‐focused support within prehabilitation influences later adjustment. Our findings concern the completeness and candour of information rather than its quantity: participants identified no optimal amount, and more is not necessarily better. Information may therefore need to be prioritised, staged and revisited, with clinicians checking what each patient wants to know and has understood, and directing them to trusted sources. The optimal timing and amount remains a question for future research.

The findings also illustrate how resilience theories [23, 35] may apply within prehabilitation, and they align with prospective longitudinal studies in cancer that examined psychological predictors of later distress [24], highlighting the role of appraisals, coping, and social resources. Participants described shifts in how they appraised treatment and their capacity to cope, consistent with self‐efficacy processes [24, 36]. Confidence was often grounded in appraisals about bodily capability shaped by visible physical progress, practising exercises, and staff encouragement. Supportive relationships, including opportunities to connect with others and to talk openly with family, strengthened motivation, reduced isolation, and helped participants stay anchored to valued roles and routines. In parallel, taking action—drawing on existing resources, identifying controllable steps, and engaging with preparation—appeared to reinforce self‐efficacy and help keep anxiety manageable.

## 5 Implications

Our findings offer a patient‐informed way to conceptualise psychological preparation in multimodal prehabilitation. They also reframe its goal: not to eliminate anxiety but to keep it manageable and compatible with approaching surgery, while remaining alert to anxiety that is severe, persistent or disabling and warrants specialist assessment. Clinically, this suggests three practical emphases.

- Reinforcing consistent expectation‐setting, prioritising complete and candid information about likely recovery trajectories (including uncertainty) rather than focussing primarily on reducing anxiety. Candid expectation‐setting is already good clinical practice; what these findings add is patient‐derived prioritisation of it, and the observation that prehabilitation offers an opportunity to revisit and personalise clinical information as concrete recovery expectations, particularly where consultations are brief or outcomes uncertain;
- Explicit relational support, recognising that readiness can be held back when patients find it difficult to tell family and friends about their diagnosis, and that brief, tailored support for disclosure and family communication, alongside peer connection, may be integral rather than optional; and
- Agency‐building, helping patients identify controllable steps, make progress visible, and link preparation to valued roles and routines.

Future research should first examine whether this provisional model transfers across services, treatment pathways and patient groups; then co‐produce readiness‐focused support with patients and clinicians; then establish acceptability and feasibility, including a feasibility study. Work to operationalise and measure readiness using patient‐informed definitions should proceed alongside. Only then would it be appropriate to test whether readiness‐focused provision within prehabilitation predicts post‐operative adjustment, using readiness‐relevant outcomes rather than distress alone.

## 6 Limitations

These findings should be interpreted in light of the study context and sample. We recruited from a single tertiary prehabilitation service focussed on major cancer surgery, so the themes reflect a specific organisational model and may not transfer directly to other services or treatment pathways. Most participants had colorectal cancer, so the themes may largely reflect colorectal surgical pathways; we did not restrict the analysis post hoc because the study aim concerned readiness across major cancer surgery, and excluding the two participants with other tumour types would have discarded relevant data and further reduced the sample. While the sample was diverse in relation to deprivation, it was small and demographically skewed (predominantly White British and largely retired), which may have limited the range of perspectives captured, particularly for patients from ethnically minoritised communities, younger patients, and those balancing work and caring responsibilities. Some interviews were brief and, although all core topic‐guide domains were addressed in the shortest interviews, this may have constrained the depth of individual accounts. In addition, participants self‐selected into interviews and all completed both time points, so views may under‐represent patients who decline prehabilitation, disengage, or experience more severe post‐operative difficulties. Because all participants were recruited from a prehabilitation service, the study cannot determine how readiness develops in its absence, nor attribute the processes we identified to prehabilitation. Patients without structured prehabilitation may have different access to professional encouragement, peer contact and opportunities for visible action, though this is an inference rather than a finding; comparative work with patients who are not offered, cannot access, decline or disengage from prehabilitation would strengthen conclusions about what is specific to it. We also did not record dates of surgery, so we cannot report how long after surgery the T2 interviews took place; interviews were scheduled around participants' recovery, and variation in this interval may have shaped how they recalled and appraised their readiness. From a critical realist standpoint, the study offers provisional, patient‐informed interpretations of the processes shaping readiness. These require examination in larger and more diverse samples across different service configurations before they can be treated as established.

## 7 Conclusions

This study provides an initial patient‐informed account of what it means to be psychologically ready for major cancer surgery within prehabilitation. Participants described readiness as a future‐focused “mindset to have surgery”, characterised by confidence, contained anxiety, and motivation to stay engaged, and shaped through reliable information, reciprocal support, and a developing sense of agency. These findings identify candidate targets for readiness‐focused, proactive psychological preparation before and during treatment: clearer and more concrete expectations, relational scaffolding, and support for patients to identify and act on what is within their control. This provisional model now requires earlier developmental work: examining transferability across settings and patient groups, co‐producing readiness‐focused support, and establishing acceptability and feasibility, before measures and interventions can be tested for their impact on readiness and post‐operative adjustment.

## Author Contributions

**Aniqa Hussain:** conceptualisation, methodology, formal analysis, investigation, data curation, visualisation, project administration, writing – original Draft. **Thomas Schroder:** conceptualisation, methodology, writing – review and editing, supervision. **Mike Rennoldson:** conceptualisation, methodology, resources, writing – review and editing, supervision. **Michael Baliousis:** conceptualisation, methodology, visualisation, resources, writing – original draft, writing – review and editing, supervision.

## Funding

The authors have nothing to report.

## Conflicts of Interest

The authors declare no conflicts of interest.

## Supporting information


Supporting Information S1

Supporting Information S2

## Data Availability

The data that support the findings of this study are available on request from the corresponding author. The data are not publicly available due to privacy or ethical restrictions.
