# Supplementary material for: Psychological Readiness for Surgery Through Prehabilitation and Treatment: A Longitudinal Qualitative Study
Source: Psychooncology. 2026 Aug 28;35(9):e70594. doi: 10.1002/pon.70594 (PMC13525233; doi:10.1002/pon.70594)
Supplement: Supplementary file 2 [file PON-35-e70594-s001.docx]

**Interview Schedule**

**(Final Version 6: 19/02/2024)**

**Interview 1**

**How did your cancer journey start?**

Prompt: Journey to diagnosis?

Prompt: Journey after diagnosis?

**We are trying to understand more about people’s experience of psychological readiness for treatment. What does psychological readiness mean to you?**

Prompt: [unpacking psychological readiness- emotional/ cognitive components]

Prompt: What feelings and thoughts do you associate with being ‘psychologically ready’ for surgery?

**How [psychologically ready]* for treatment would you consider yourself currently?**

*use the participant’s language for psychological readiness

**Have you always felt this way, or has your sense of readiness changed over time?**

Prompt: Has your sense of psychological readiness changed since diagnosis?

**What has contributed to you feeling this way?**

Prompt: Is there anything you did to help prepare yourself for treatment?

Prompt: [What and who contributed? How did this affect them and their journey?]

**Have you been using methods or learnings from your experience of challenging situations in the past? And have these been effective in psychologically readying you for treatment?**

Prompt: [unpack similarities/differences]

Prompt: [connections to sense of readiness now]

Prompt: Have you used learning from witnessing how other people have managed challenging situations?

Prompt: [Any previous experiences of therapy?]

**What have you found helpful in helping you feel psychologically ready for surgery so far?**

Prompt: key relationships?

**What have you found unhelpful?**

Prompt: [unhelpful attitudes/discourse?]

**What might help you feel more psychologically prepared for surgery?**

Prompt: [helpful attitudes/discourse?]

**What might you like our team to offer you to support your psychological readiness for treatment?**

Prompt**:** What are your expectations for prehab in terms of helping you prepare psychologically for your treatment?

**Interview 2**

**What was the outcome of your surgery? Did it progress as you expected it to?**

**Has your understanding of psychological readiness changed since completing prehabilitation?**

[Remind participant of their answer from the first interview]

Prompt: [unpacking psychological readiness- emotional/ cognitive components]

**Has your understanding of psychological readiness changed now you have had the surgery?**

**How psychologically ready did you consider yourself for surgery?**

Prompt: Did you feel psychologically prepared?

Prompt: Do you feel more could have been done to psychologically prepare you?

**Looking back, how do you feel prehabilitation contributed to your psychological readiness for treatment, if at all?**

**Looking back, what did you find helpful?**

Prompt: What methods did you find helpful?

Prompt: What and who helped you feel more psychologically prepared?

Prompt: Did the team do anything that was particularly helpful?

**Looking back, what did you find unhelpful?**

Prompt: What methods did you find unhelpful?

Prompt: Is there something the team could have done differently, to help you feel more prepared?

**Since surgery, has your view of cancer treatment changed at all?**

Prompt: Did prehabilitation change your view of treatment?

**In what ways did prehabilitation meet or not meet your expectations in helping you prepare psychologically?**

**What could be different in your prehabilitation journey, to help you prepare better psychologically for treatment?**
